# Sleep Trajectories and All-Cause Mortality Among Low-Income Adults

**DOI:** 10.1001/jamanetworkopen.2024.62117

**Published:** 2025-02-27

**Authors:** Kelsie M. Full, Hui Shi, Loren Lipworth, Lawrence T. Dauer, Michael T. Mumma, Qian Xiao

**Affiliations:** 1 Division of Epidemiology, Department of Medicine, Vanderbilt University Medical Center, Nashville, Tennessee; 2 Vanderbilt Memory and Alzheimer’s Center, Vanderbilt University Medical Center, Nashville, Tennessee; 3 Memorial Sloan Kettering Cancer Center, New York, New York; 4 Department of Epidemiology, Human Genetics, and Environmental Sciences, School of Public Health, The University of Texas Health Science Center at Houston, Houston; 5 Center for Spatial-Temporal Modeling for Applications in Population Sciences, School of Public Health, The University of Texas Health Science Center at Houston, Houston

## Abstract

**Importance:**

Short and long sleep durations are adversely associated with cardiovascular disease (CVD), type 2 diabetes, and mortality. It remains unclear how sleep duration trajectories over time are associated with mortality and whether these associations vary by well-documented sex, race, and socioeconomic sleep disparities.

**Objective:**

To investigate the association of 5-year sleep duration trajectories with all-cause and cause-specific mortality among US adults, predominantly those in low-income groups.

**Design, Setting, and Participants:**

The Southern Community Cohort Study included participants aged 40 to 79 years recruited and enrolled (from March 2002 to September 2009) from community health centers by using random sampling methods across 12 states in the Southeastern US. Participants completed a follow-up survey between 2008 and 2013. Data analysis was performed from August 10 to November 30, 2023.

**Exposures:**

Sleep duration was self-reported at study enrollment and at 5-year follow-up. At each time point, sleep was categorized as short (<7 hours), healthy (7-9 hours), or long (>9 hours). Nine sleep trajectories were defined based on 5-year change or consistency in sleep duration category between enrollment and follow-up.

**Main Outcome and Measures:**

Cause of death was ascertained via linkage to the National Death Index through December 31, 2022. Multivariable-adjusted Cox proportional hazards regression analysis was performed to estimate hazard ratios (HRs) and 95% CIs for mortality outcomes (all-cause, CVD, cancer, and neurodegenerative disease) associated with sleep duration trajectory.

**Results:**

Participants included 46 928 adults (mean [SD] age, 53.0 [8.8] years; 65.4% women; 63.3% self-identified as Black and 36.7% as White; and 47.5% with a household income <$15 000 per year). Overall, 66.4% of participants had suboptimal 5-year sleep trajectories. Race varied across sleep trajectories; 53.0% of participants in the optimal trajectory were Black, compared with 84.5% in the long-short trajectory. During a median 12.6 (IQR, 11.3-13.1) years of follow-up, 13 579 deaths occurred (4135 from CVD, 3067 from cancer, and 544 from neurodegenerative diseases). Compared with the optimal sleep duration trajectory, suboptimal trajectories were associated with as much as 29% greater risk of all-cause mortality in fully-adjusted models. For all-cause and CVD-specific mortality, the long-long (HRs, 1.27 [95% CI, 1.14-1.41] and 1.22 [95% CI, 1.01-1.48], respectively) short-long (HRs, 1.29 [95% CI, 1.17-1.42] and 1.22 [95% CI, 1.03-1.45], respectively), and long-short (HRs, 1.19 [95% CI, 1.05-1.35] and 1.32 [95% CI, 1.07-1.63], respectively) trajectories were associated with the greatest risk. After adjustment for comorbid conditions, no associations were observed for mortality due to cancer or neurodegenerative disease. Observed associations varied by race and household income, with the greatest risk observed among White adults with greater household incomes.

**Conclusions and Relevance:**

In this cohort study of 46 928 US residents, nearly two-thirds of participants had suboptimal 5-year sleep duration trajectories. Suboptimal sleep duration trajectories were associated with as much as a 29% increase in risk of all-cause mortality. These findings highlight the importance of maintaining healthy sleep duration over time to reduce mortality risk.

## Key Points

**Question:**

Are 5-year sleep duration trajectories associated with all-cause and cause-specific mortality outcomes among Black and White US residents from lower household income groups?

**Findings:**

In this community-based cohort study of 46 928 adults, approximately two-thirds of Black and White adults had suboptimal 5-year sleep duration trajectories. Black participants constituted 53% of those in the optimal trajectory and 84% in the long-short trajectory. Suboptimal sleep trajectories were associated with up to a 29% greater risk of mortality, with variations in associations by race and household income.

**Meaning:**

These findings highlight the importance of maintaining a healthy sleep duration over time to reduce mortality risk.

## Teaser

This cohort study investigates the association of 5-year sleep duration trajectories with the risks of all-cause and cause-specific mortality among US Black and White adults with lower income.

## Introduction

The American Academy of Sleep Medicine^1^ and National Sleep Foundation^2^ recommend adults sleep 7 hours to 9 hours per night to maintain optimal overall health.^3^ Only 30% to 45% of adults in the US meet these recommendations.^4^ Differences in meeting sleep duration recommendations have been observed by race and ethnicity, socioeconomic status (SES), and sex and gender. In a National Health and Nutrition Examination Survey study,^5^ 35% of participants reported sleeping 6 or fewer hours per night. Compared with other racial and ethnic groups, the highest prevalence of short sleep duration was reported among non-Hispanic Black adults,^5^ consistent with well-documented racial and ethnic sleep disparities.^6,7^ In a cohort of middle-aged adults, Black participants had shorter sleep duration and lower sleep efficiency compared with Asian and non-Hispanic White adults.^8^ Differences were also observed by SES, with lower SES associated with several measures of poor sleep quality.^8^ Sex and gender differences in sleep health have also been observed.^9^ The age-adjusted prevalence of reporting difficulty sleeping increased from 2005 to 2018 in the overall population, including among both sexes and all racial and ethnic groups,^5^ highlighting the need for public health strategies to address poor sleep health at the population level.

Abnormal sleep duration has been associated with adverse health outcomes, including cardiovascular disease (CVD),^10,11,12^ cancer, and mortality.^13,14^ A U-shaped association between sleep duration and mortality has been consistently observed, indicating that both short and long sleep durations are associated with increased risk of death.^13,14,15^ Among 407 500 adults in the UK Biobank cohort,^13^ self-reported short (≤5 hours) and long (≥9 hours) sleep durations were associated with a 25% to 30% higher risk of all-cause mortality and a 27% to 32% higher risk of CVD mortality. Long sleep duration was also associated with a higher risk of cancer mortality.^13^ Most prior studies investigating sleep duration and mortality have only measured sleep duration at a single time point.^14^ Evidence suggests that the association between sleep and health outcomes, such as CVD risk factors, vary by sex, self-identified race and ethnicity, and SES.^16^ Less is known about how changes in sleep duration over time relate to mortality and whether associations vary by sex, race and ethnicity, and SES.

We investigated the association of sleep duration trajectories over time with the risk of all-cause and cause-specific mortality in the Southern Community Cohort Study (SCCS), a large US cohort primarily including Black and White adults representing lower household incomes. We leveraged repeat measures of sleep duration within a cohort large enough to investigate differences by sex, race and ethnicity, and SES. Few prior studies are appropriately designed to simultaneously investigate the potential interaction of race and SES on the association of sleep duration and mortality.^16^ We hypothesized that sleep duration trajectories that differ from maintaining an optimal healthy sleep duration of 7 to 9 hours per night would be associated with higher risk of mortality. We also hypothesized that differences would be observed in the association between Black and White adults and across sex and SES subgroups.

## Methods

The SCCS is a prospective cohort study designed to assess chronic disease disparities among adults in 12 Southeastern US states. Nearly 86 000 adults were recruited and enrolled in the SCCS from March 2002 to September 2009.^17^ To be eligible, adults had to be 40 to 79 years of age, speak English, and not have been treated for cancer in the 12 months before enrollment. Approximately 86% of enrolled participants were recruited from community health centers that provide basic health services, mainly to persons who qualify with lower incomes and no insurance, whereas the remaining 14% were randomly recruited through general population sampling. Detailed descriptions of SCCS methods have been published.^17,18,19^ Written informed consent was obtained and study protocols were approved by institutional review boards at Vanderbilt University Medical Center and Meharry Medical College, Nashville, Tennessee. This report follows the Strengthening the Reporting of Observational Studies in Epidemiology (STROBE) reporting guidelines.

At enrollment, SCCS participants completed a questionnaire on sociodemographic characteristics, health behaviors, and health history, as well as an 89-item food frequency questionnaire. Between 2008 and 2013, participants were asked to complete a follow-up survey. The present study includes Black and White participants who completed the enrollment and follow-up survey. The analysis was restricted to self-reported Black and non-Hispanic White participants, as limited numbers in other racial groups prevented stable statistical analysis.

### Mortality

Participant vital status and date and cause of death were ascertained via linkage to the National Death Index (NDI) through December 31, 2022. Cause-specific categories of death were classified according to NDI recode values, including major cardiovascular diseases (CVD; 053), malignant neoplasms (cancer; 019), and neurodegenerative disease (combined Parkinson disease [051], Alzheimer disease [052]), as well as *International Statistical Classification of Diseases, Tenth Revision* codes for dementia (F00, F01, and F03).

### Sleep Trajectories

At enrollment, participants were asked “how many hours do you typically sleep in a 24-hour period?” with weekdays and weekends reported separately. Baseline sleep duration was calculated as the weighted mean of weekday and weekend sleep [([weekday duration × 5] + [weekend duration × 2]) / 7]. In the follow-up survey, participants were asked, “In a 24-hour period, how many hours do you typically spend sleeping?” with no distinction between weekday and weekend sleep.

Sleep duration was categorized as short (<7 hours), healthy (7-9 hours), and long (>9 hours) based on current recommendations.^20^ We defined 9 sleep trajectories based on change or consistency in sleep duration category between enrollment and follow-up. The healthy-healthy category served as the reference group for all analyses (optimal; maintaining a sleep duration of 7-9 hours across the 5-year period). Suboptimal categories included transition from healthy sleep duration to short (healthy-short) or long (healthy-long) durations, maintaining short sleep durations (short-short), transition from short to healthy (short-healthy) or short to long (short-long) durations, maintaining long durations (long-long), or transition from long to healthy (long-healthy) or long to short (long-short) durations.

### Covariates

Relevant covariates were obtained from the enrollment questionnaire. Sociodemographic variables included age at enrollment, sex, self-reported race (Black or White), educational attainment (some high school or less or high school diploma or beyond), marital status (married or living as married with a partner or separated, divorced, widowed, or single), household income (≥$15 000 or <$15 000), and employment status (unemployed or employed). Health behaviors included smoking status (current or noncurrent smoker), alcohol intake (≤1 or >1 drink/d), diet quality, physical activity, and sedentary time. Diet quality was estimated with the updated Healthy Eating Index.^21^ Total moderate physical activity (metabolic equivalent task–hours) was the sum of moderate occupation and sports-related activity energy expenditure, categorized into quartiles. Sedentary time was the total reported hours spent sitting, categorized into quartiles. Body mass index was calculated as weight in kilograms divided by height in meters squared. Depressive symptoms were assessed with the validated Center for Epidemiological Studies Depression scale.^22^ Comorbid conditions were incorporated with a modified version of the Charlson Comorbidity Index (CCI), which provides a weighted value that is highly predictive of mortality due to a comorbid condition.^23^ The modified CCI includes a self-reported history of physician diagnosis of myocardial infarction (MI), stroke, chronic obstructive pulmonary disorder, asthma or tuberculosis, hepatitis, ulcer, diabetes, Parkinson disease, hypertension or hypercholesterolemia, arthritis, inflammatory bowel disease, multiple sclerosis, lupus, cancer, and HIV-AIDS.

### Statistical Analyses

Analyses were performed from August 10 to November 30, 2023. Participants’ characteristics were described by sleep duration trajectory category. Descriptive statistics are presented as mean (SD) or median (IQR) for continuous variables and frequency (percentage) for categorical variables. To visualize the association of sleep trajectory and all-cause mortality, we used a Kaplan-Meier plot adjusted for age, sex, and race and calculated the cumulative risk of cause-specific mortality by sleep trajectory, taking into account competing risks.

Survival time was calculated as the time between date of first follow-up survey and date of death or December 31, 2022. To examine the association between sleep trajectory and all-cause mortality, we ran a series of sequentially adjusted Cox proportional hazards regression models. Next, adjusted Fine-Gray competing risks regression models were used for CVD-, cancer-, and neurodegenerative disease–specific mortality outcomes. Model 1 was adjusted for age, sex, and race. Model 2 added adjustment for individual SES variables. In our primary model, model 3, we further adjusted for health behaviors, including smoking status, alcohol intake, diet quality, moderate physical activity, and sedentary time. The fully adjusted model (model 4) included adjustment for body mass index, depressive symptoms, and the CCI. Each model was restricted to complete covariate data. Model fit was sequentially evaluated with Akaike information criterion, Bayes information criterion, and the C index.

We conducted several sensitivity analyses. We tested for effect modification by sex, race, and SES indicators, including educational attainment and household income. Cut points for educational attainment and income were chosen to create equal sample sizes in subgroups to preserve statistical power. Preliminary analyses showed evidence of an interaction between sleep trajectory and race and between sleep trajectory and household income; therefore, we reran analyses stratified by race and household income. We repeated all analyses excluding participants who reported an MI at enrollment or follow-up (n = 5373). We repeated all models after excluding participants who died within 2 years of follow-up to address possible reverse causation. Missingness in covariates ranged from 0 to 5% (eFigure 2 in Supplement 1). To explore whether associations were impacted by missing data, we used multiple imputation by chained equations to impute missing values of covariates with 20 imputations and reran primary models.

We evaluated the proportional hazards assumption using the Schoenfeld residuals method and log-log (survival) vs log-time plots. The assumptions did not hold for all variables in the primary analysis (*P* < .05), with evidence of minimal deviation for the sleep duration trajectories. Two-sided *P* < .05 indicated statistical significance. All statistical analyses were performed in 2023 using R statistical software, version 4.3.1 (R Foundation).^24^

## Results

A total of 50 644 patients were eligible for participation. We excluded those who were missing information on sleep duration at enrollment or follow-up (n = 3679) and those who were not followed up past the follow-up survey (n = 37). The analytic sample included 46 928 participants (eFigure 1 in Supplement 1). The mean (SD) age of participants at SCCS enrollment was 53.0 (8.8) years; 34.6% were men and 65.4% were women; 63.3% self-identified as Black and 36.7% as White; and 47.5% reported a household income less than $15 000 per year (eTable 1 in Supplement 1). Mean (SD) sleep duration at enrollment was 7.2 (1.9) hours per night.

Participant baseline characteristics according to sleep trajectory category are presented in Table 1. A total of 15 781 participants (33.6%) maintained an optimal 5-year sleep trajectory within the recommended 7 to 9 hours per night, and 31 147 (66.4%) did not. Participants with an optimal trajectory were older and more likely to have educational attainment beyond a high school diploma and a household income of $50 000 or greater. The most common suboptimal sleep trajectories among participants were short-short (n = 9486), short-healthy (n = 7878), and healthy-short (n = 4900). Participants with a short-short or a healthy-short trajectory were more likely to be women. Race appeared to vary across sleep trajectories, as 53.0% of participants in the optimal trajectory compared with 84.5% of participants in the long-short trajectory identified as Black. Household income also varied across the trajectories, with a greater proportion of participants in the long-short and short-long trajectories having a household income less than $15 000 compared with other trajectories.

**Table 1.** Southern Community Cohort Study Participant Characteristics Stratified by Sleep Duration Trajectory

Characteristic	Sleep trajectory group, No. (%)^a^								
	Optimal (n = 15 781)	Long-long (n = 1283)	Long-healthy (n = 2581)	Long-short (n = 990)	Healthy-long (n = 2401)	Healthy-short (n = 4900)	Short-long (n = 1628)	Short-healthy (n = 7878)	Short-short (n = 9486)
Age, mean (SD), y	54.3 (9.1)	52.3 (8.5)	51.9 (8.5)	51.3 (8.2)	53.7 (9.3)	52.5 (8.8)	52.1 (8.4)	52.8 (8.5)	52.0 (8.1)
Sex									
Men	5729 (36.3)	460 (35.9)	924 (35.8)	348 (35.2)	900 (37.5)	1588 (32.4)	555 (34.1)	2689 (34.1)	3022 (31.9)
Women	10 052 (63.7)	823 (64.1)	1657 (64.2)	642 (64.8)	1501 (62.5)	3312 (67.6)	1073 (65.9)	5189 (65.9)	6464 (68.1)
Self-identified race									
Black	8366 (53.0)	895 (69.8)	1981 (76.8)	837 (84.5)	1605 (66.8)	3505 (71.5)	1145 (70.3)	5109 (64.9)	6283 (66.2)
White	7415 (47.0)	388 (30.2)	600 (23.2)	153 (15.5)	796 (33.2)	1395 (28.5)	483 (29.7)	2769 (35.1)	3203 (33.8)
Educational attainment									
Less than high school	3112 (19.7)	341 (26.6)	731 (28.4)	306 (30.9)	710 (29.6)	1280 (26.1)	541 (33.3)	1903 (24.2)	2205 (23.3)
High school diploma	4648 (29.5)	453 (35.3)	918 (35.6)	351 (35.5)	818 (34.1)	1606 (32.8)	566 (34.8)	2519 (32.0)	2990 (31.5)
Beyond high school	8007 (50.8)	489 (38.1)	928 (36.0)	332 (33.6)	872 (36.3)	2012 (41.1)	520 (32.0)	3448 (43.8)	4284 (45.2)
Married	7423 (47.8)	410 (32.2)	902 (35.2)	345 (35.1)	866 (36.3)	1853 (38.1)	503 (31.1)	3072 (39.3)	3595 (38.2)
Household income, US $									
<15 000	6186 (39.9)	784 (61.8)	1461 (57.4)	624 (63.9)	1394 (59.1)	2501 (51.7)	1034 (64.3)	3746 (48.2)	4564 (48.8)
15 000 to <50 000	6144 (39.7)	408 (32.2)	909 (35.7)	310 (31.8)	808 (34.2)	1818 (37.6)	505 (31.4)	3057 (39.4)	3645 (38.9)
≥50 000	3155 (20.4)	76 (6.0)	177 (6.9)	42 (4.3)	156 (6.6)	517 (10.7)	70 (4.4)	963 (12.4)	1151 (12.3)
Unemployed	8071 (51.7)	976 (77.2)	1644 (64.9)	675 (68.9)	1603 (67.9)	2735 (56.4)	1110 (69.5)	4192 (53.8)	5153 (54.9)
Current smoker	4349 (27.8)	528 (41.5)	1076 (42.2)	437 (44.5)	873 (36.7)	1713 (35.2)	700 (43.3)	2668 (34.1)	3395 (36.1)
Alcohol consumption >1 drink/d	2661 (17.2)	257 (20.4)	537 (21.3)	225 (23.0)	445 (18.8)	874 (18.1)	309 (19.2)	1372 (17.7)	1572 (16.8)
HEI-2010 score, median (IQR)^b^	60.4 (51.5-69.6)	56.7 (49.4-64.0)	57.2 (49.3-65.4)	56.8 (48.5-63.8)	57.1 (48.9-65.5)	58.7 (50.2-67.0)	56.2 (48.2-64.9)	58.4 (50.0-67.4)	58.4 (49.5-67.2)
Physical activity, median (IQR), MET-h/d	8.0 (4.0-12.6)	6.9 (2.9- 12.0)	8.0 (4.0- 13.1)	8.0 (3.9- 13.7)	8.0 (4.0- 12.6)	8.0 (4.0- 13.7)	6.9 (2.9- 12.1)	8.0 (4.0- 13.1)	8.0 (4.0-13.7)
Sedentary time, median (IQR), h/d	8.3 (6.0-11.8)	9.5 (6.3- 13.2)	10.0 (6.5- 13.3)	9.1 (6.0- 14.0)	8.5 (6.0- 12.3)	8.5 (6.0- 12.0)	8.8 (6.0- 13.0)	8.5 (6.0- 12.0)	9.0 (6.0-12.3)
BMI, median (IQR)	29.1 (25.1-34.0)	30.1 (25.8-36.0)	29.9 (25.1-35.3)	30.6 (25.7-36.2)	29.5 (25.2-35.3)	30.0 (25.7-35.0)	30.3 (25.8-36.0)	29.8 (25.7-35.3)	30.1 (25.8-35.6)
CES-D score, mean (SD)^c^	6.7 (5.2)	10.2 (6.5)	8.9 (5.9)	9.9 (6.0)	8.8 (5.9)	8.1 (5.6)	11.6 (6.6)	9.2 (6.1)	10.0 (6.4)
CCI score, mean (SS)	1.8 (1.4)	2.2 (1.6)	2.0 (1.5)	2.1 (1.5)	2.1 (1.5)	1.9 (1.5)	2.3 (1.6)	2.0 (1.5)	2.1 (1.5)
MI at follow-up 1	502 (3.2)	64 (5.1)	112 (4.5)	51 (5.3)	130 (5.5)	190 (4.0)	96 (6.1)	328 (4.3)	453 (4.9)

Abbreviations: BMI, body mass index (calculated as the weight in kilograms divided by the height in meters squared); CES-D, Center for Epidemiological Studies Depression Scale; CCI, Charlson Comorbidity Index; HEI-2010, updated Healthy Eating Index; MET, metabolic equivalent task; MI, myocardial infarction.

^a^ Includes 46 928 participants. Owing to missing data, denominators may differ from numbers in column headings. Sleep trajectories were categorized during a 5-year period as change from or consistently healthy (7-9 hours/night), short (<7 hours/night), or long (>9 hours/night). Optimal indicates a healthy-healthy trajectory.

^b^ Scores range from 0 to 100, with higher scores indicating better diet quality.

^c^ Scores range from 0 to 60, with higher scores indicating greater depressive symptoms.

### All-Cause Mortality

Over a median follow-up of 12.6 (IQR, 11.3-13.1) years, 13 579 deaths occurred, including 4135 from CVD, 3067 from cancer, and 544 from neurodegenerative disease. The risk of mortality differed significantly across sleep trajectory categories (overall *P* < .001) (eFigure 3 in Supplement 1).

Compared with those with an optimal sleep trajectory, participants with every other sleep trajectory had a greater risk of all-cause mortality (Table 2). After adjustment for demographics, socioeconomic factors, and health behaviors (model 3), all suboptimal trajectories were associated with an HR for mortality ranging from 1.09 (95% CI, 1.03-1.15) for short-short to 1.40 (95% CI, 1.28-1.53) for short-long trajectories. After further adjustment for depressive symptoms and comorbid conditions, all associations for suboptimal sleep trajectories persisted except for the association observed among the short-short trajectory. The short-long (HR, 1.29; 95% CI, 1.17-1.42), long-short (HR, 1.19; 95% CI, 1.05-1.35), and long-long (HR, 1.27; 95% CI, 1.14-1.41) sleep trajectories were associated with the greatest risk of mortality compared with the optimal trajectory. The fully adjusted model (model 4) had the lowest Akaike information criterion and Bayes information criterion and the highest C index (eTable 2 in Supplement 1).

**Table 2.** Sleep Trajectories and All-Cause Mortality in the Southern Community Cohort Study^a^

Sleep trajectory	No. of participants	No. of events	HR (95% CI)			
			Model 1^b^	Model 2^c^	Model 3^d^	Model 4^e^
Optimal	15 781	4197	1 [Reference]	1 [Reference]	1 [Reference]	1 [Reference]
Long-long	1283	457	1.62 (1.47-1.78)	1.32 (1.20-1.46)	1.31 (1.18-1.46)	1.27 (1.14-1.41)
Long-healthy	2581	849	1.51 (1.40-1.62)	1.30 (1.20-1.40)	1.25 (1.15-1.36)	1.19 (1.09-1.29)
Long-short	990	335	1.61 (1.44-1.80)	1.33 (1.19-1.49)	1.26 (1.11-1.42)	1.19 (1.05-1.35)
Healthy-long	2401	862	1.56 (1.45-1.67)	1.33 (1.23-1.43)	1.28 (1.18-1.38)	1.22 (1.13-1.33)
Healthy-short	4900	1439	1.27 (1.20-1.35)	1.16 (1.09-1.23)	1.14 (1.07-1.22)	1.11 (1.04-1.19)
Short-long	1628	608	1.83 (1.68-2.00)	1.48 (1.35-1.61)	1.40 (1.28-1.53)	1.29 (1.17-1.42)
Short-healthy	7878	2281	1.22 (1.16-1.29)	1.15 (1.09-1.21)	1.13 (1.07-1.19)	1.08 (1.02-1.14)
Short-short	9486	2551	1.19 (1.13-1.25)	1.12 (1.06-1.18)	1.09 (1.03-1.15)	1.02 (0.96-1.07)

Abbreviation: HR, hazard ratio.

^a^ Includes 46 928 participants. Sleep trajectories were categorized during a 5-year period as change from or consistently healthy (7-9 hours/night), short (<7 hours/night), or long (>9 hours/night). Optimal indicates a healthy-healthy trajectory.

^b^ Adjusted for age, sex, race.

^c^ Adjusted for covariates in model 1 plus educational attainment, marital status, household income, and employment status.

^d^ Adjusted for covariates in model 2 plus smoking status, alcohol intake, updated Healthy Eating Index, total moderate activity, and sedentary time.

^e^ Adjusted for covariates in model 3 plus body mass index, Center for Epidemiological Studies Depression Scale score, and Charlson Comorbidity Index.

### Cause-Specific Mortality

**Figure 1.**
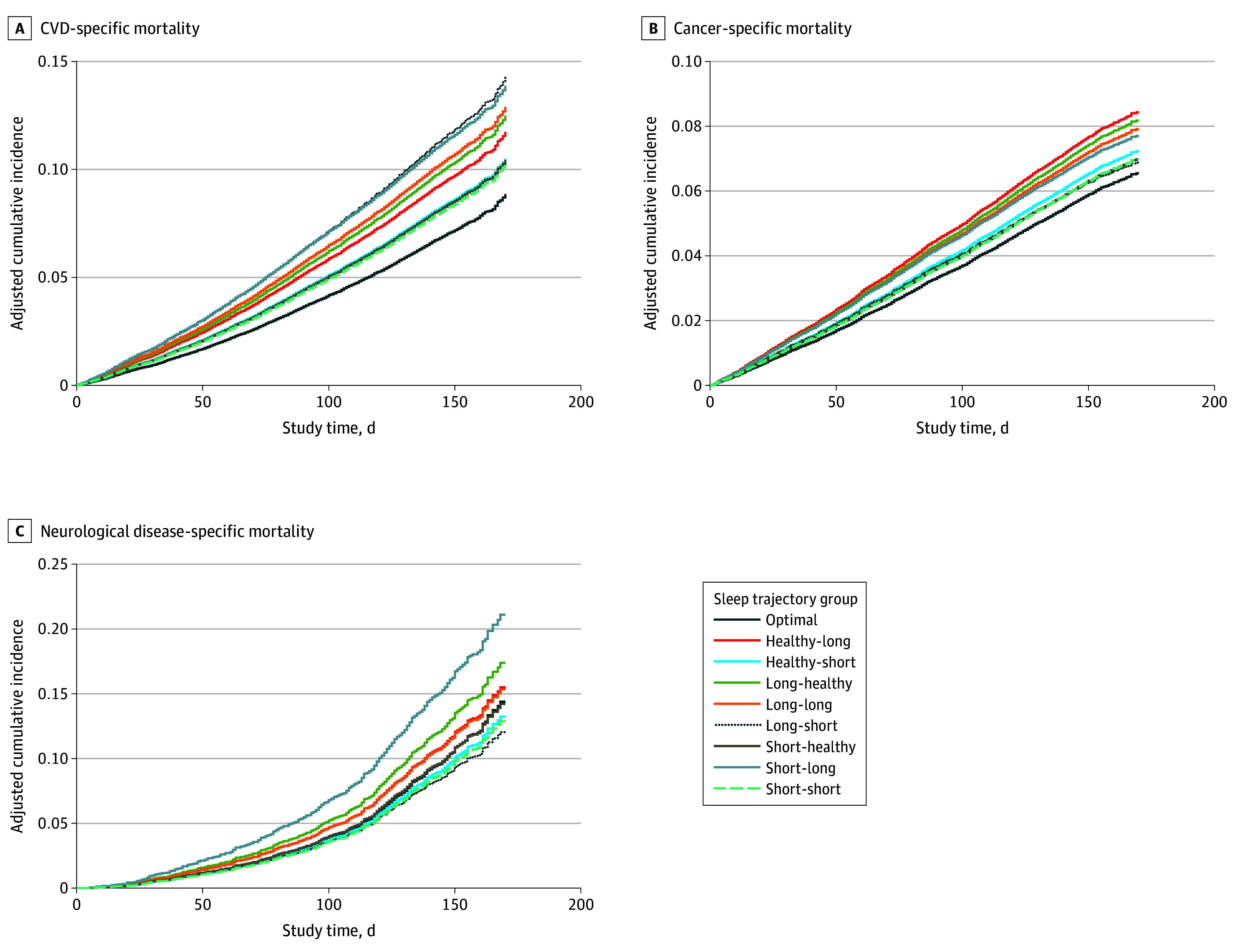
Cumulative Risk of Cause-Specific Mortality by Sleep Trajectory, Adjusting for Competing Risks Data are from the Southern Community Cohort Study from 2008 to 2022. Sleep trajectories were categorized during a 5-year period as change from or consistently healthy (7-9 h/night), short (<7 h/night), or long (>9 h/night). Optimal indicates a healthy-healthy trajectory. CVD indicates cardiovascular disease.

Figure 1 depicts the cumulative risk of cause-specific mortality by sleep trajectory, adjusting for the competing risk of death. Compared with participants who maintained an optimal sleep trajectory, almost all other sleep trajectories were associated with greater risk of CVD mortality (Table 3 and eFigure 4 in Supplement 1). In model 3, the greatest risk of CVD mortality was again observed among the long-short (HR, 1.40; 95% CI, 1.14-1.71) and the short-long (HR, 1.32; 95% CI, 1.12-1.57) trajectories. The observed associations persisted after adjustment for the CCI for the long-long (HR, 1.22; 95% CI, 1.01-1.48), long-healthy (HR,1.16; 95% CI, 1.00-1.34), long-short (HR, 1.32; 95% CI, 1.07-1.63), and short-long (HR, 1.22; 95% CI, 1.03-1.45) trajectories. The only adverse association observed with cancer mortality was among participants in the healthy-long sleep trajectory (HR, 1.21; 95% CI, 1.02-1.43), and this association did not persist after adjustment for depressive symptoms and comorbid conditions. While there were no associations observed between any suboptimal sleep trajectories and mortality from neurodegenerative disease, an adverse outcome was observed for the short-long trajectory that stood out from the other trajectories (HR for the fully adjusted model, 1.46; 95% CI, 0.91-2.33).

**Table 3.** Sleep Trajectories and Cause-Specific Mortality in the Southern Community Cohort Study^a^

Sleep trajectory	No. of events	HR (95% CI)			
		Model 1^b^	Model 2^c^	Model 3^d^	Model 4^e^
**CVD mortality (n = 4135)**					
Optimal	1256	1 [Reference]	1 [Reference]	1 [Reference]	1 [Reference]
Long-long	142	1.55 (1.30-1.85)	1.31 (1.10-1.57)	1.29 (1.07-1.56)	1.22 (1.01-1.48)
Long-healthy	271	1.47 (1.29-1.68)	1.27 (1.10-1.45)	1.24 (1.07-1.43)	1.16 (1.00-1.34)
Long-short	118	1.69 (1.39-2.04)	1.43 (1.18-1.73)	1.40 (1.14-1.71)	1.32 (1.07-1.63)
Healthy-long	253	1.35 (1.18-1.55)	1.20 (1.04-1.38)	1.18 (1.02-1.37)	1.15 (0.99-1.34)
Healthy-short	435	1.20 (1.08-1.34)	1.11 (0.99-1.24)	1.10 (0.97-1.23)	1.06 (0.94-1.19)
Short-long	187	1.66 (1.42-1.94)	1.38 (1.18-1.63)	1.32 (1.12-1.57)	1.22 (1.03-1.45)
Short-healthy	695	1.20 (1.10-1.32)	1.14 (1.03-1.25)	1.12 (1.01-1.24)	1.08 (0.97-1.20)
Short-short	778	1.18 (1.07-1.29)	1.11 (1.01-1.22)	1.09 (0.99-1.20)	1.02 (0.92-1.13)
**Cancer mortality (n = 3067)**					
Optimal	1013	1 [Reference]	1 [Reference]	1 [Reference]	1 [Reference]
Long-long	93	1.23 (0.99-1.52)	1.09 (0.87-1.36)	1.05 (0.83-1.33)	1.02 (0.80-1.30)
Long-healthy	190	1.26 (1.08-1.48)	1.19 (1.02-1.40)	1.17 (0.99-1.38)	1.13 (0.95-1.35)
Long-short	60	1.06 (0.82-1.38)	0.97 (0.74-1.26)	0.90 (0.68-1.20)	0.91 (0.68-1.21)
Healthy-long	192	1.29 (1.10-1.50)	1.20 (1.03-1.42)	1.21 (1.02-1.43)	1.15 (0.97-1.37)
Healthy-short	321	1.11 (0.97-1.26)	1.05 (0.92-1.20)	1.04 (0.90-1.19)	1.03 (0.89-1.18)
Short-long	111	1.18 (0.97-1.44)	1.03 (0.84-1.27)	0.98 (0.79-1.21)	0.96 (0.77-1.19)
Short-healthy	506	1.07 (0.96-1.20)	1.04 (0.93-1.16)	1.02 (0.91-1.14)	1.01 (0.89-1.14)
Short-short	581	1.07 (0.96-1.19)	1.05 (0.94-1.17)	1.00 (0.90-1.12)	0.99 (0.88-1.11)
**Neurodegenerative disease mortality (n = 544)**					
Optimal	220	1 [Reference]	1 [Reference]	1 [Reference]	1 [Reference]
Long-long	<10	1.20 (0.70-2.03)	1.22 (0.72-2.08)	1.24 (0.71-2.16)	1.21 (0.68-2.16)
Long-healthy	28	1.21 (0.81-1.81)	1.10 (0.72-1.68)	1.08 (0.69-1.69)	1.10 (0.70-1.73)
Long-short^f^	<10	NR	NR	NR	NR
Healthy-long	35	1.09 (0.76-1.57)	1.08 (0.74-1.56)	1.12 (0.77-1.65)	1.05 (0.70-1.58)
Healthy-short	50	0.95 (0.70-1.29)	0.96 (0.71-1.31)	1.00 (0.73-1.38)	1.00 (0.72-1.39)
Short-long	24	1.59 (1.03-2.45)	1.46 (0.93-2.29)	1.47 (0.93-2.34)	1.46 (0.91-2.33)
Short-healthy	86	1.02 (0.80-1.32)	1.01 (0.78-1.31)	1.06 (0.81-1.39)	1.03 (0.78-1.37)
Short-short	79	0.92 (0.71-1.19)	0.95 (0.73-1.23)	0.95 (0.72-1.26)	0.95 (0.71-1.27)

Abbreviations: HR, hazard ratio; NR, not reportable.

^a^ Includes 46 928 participants. Sleep trajectories were categorized during a 5-year period as change from or consistently healthy (7-9 hours/night), short (<7 hours/night), or long (>9 hours/night). Optimal indicates a healthy-healthy trajectory.

^b^ Adjusted for age, sex, race.

^c^ Adjusted for covariates in model 1 plus educational attainment, marital status, household income, and employment status.

^d^ Adjusted for covariates in model 2 plus smoking status, alcohol intake, updated Healthy Eating Index, total moderate activity, and sedentary time.

^e^ Adjusted for covariates in model 3 plus body mass index, Center for Epidemiological Studies Depression Scale score, and Charlson Comorbidity Index.

^f^ NR due to partnership with Centers for Medicare & Medicaid Services and obligations to not report values less than 10.

**Figure 2.**
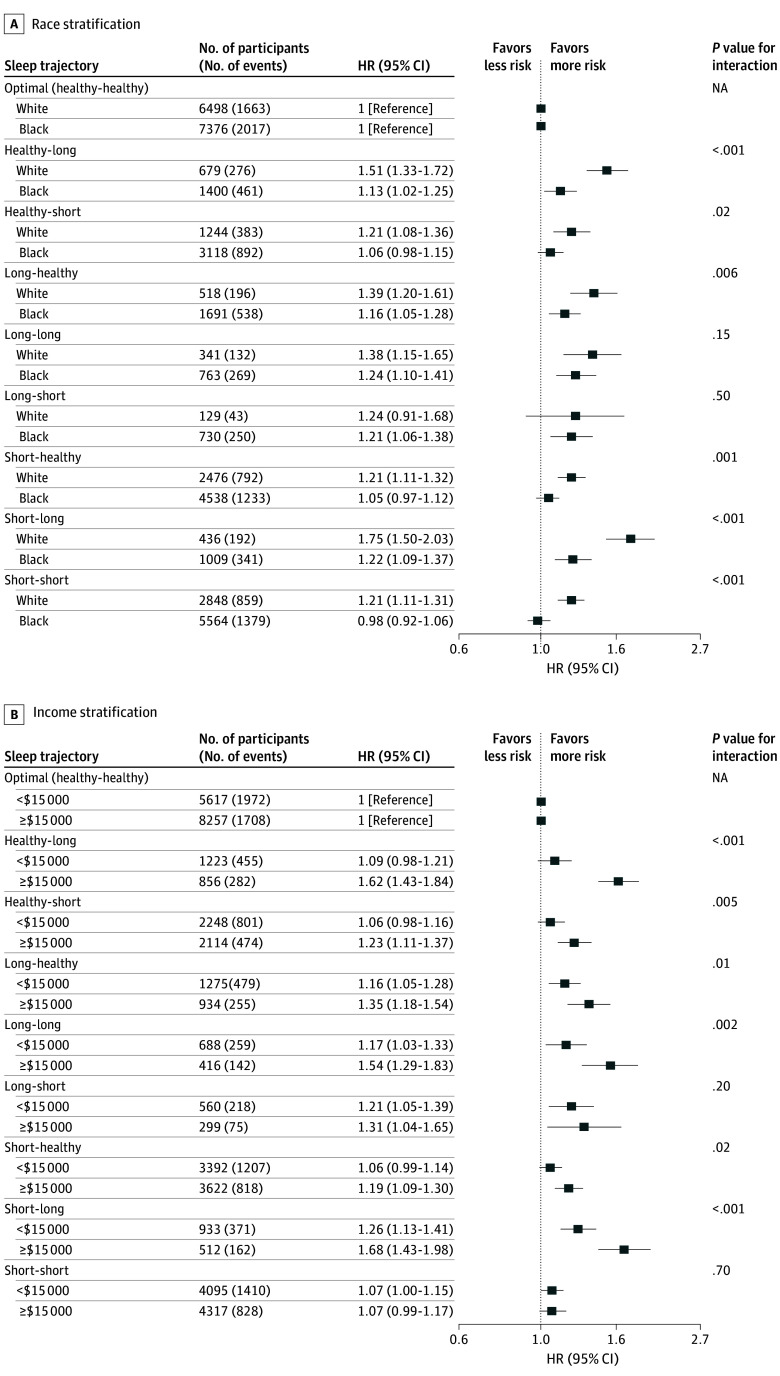
Race- and Income-Stratified Associations Between Sleep Trajectories and All-Cause Mortality Adjusted for age, sex, educational attainment, marital status, household income, employment status, smoking status, alcohol intake, updated Healthy Eating Index, total moderate activity, and sedentary time. Sleep trajectories were categorized during a 5-year period as change from or consistently healthy (7-9 h/night), short (<7 h/night), or long (>9 h/night). HR indicates hazard ratio; NA, not applicable.

In sensitivity analyses, we observed evidence of effect modification by race and income. The association between sleep trajectory and all-cause mortality significantly differed among Black and White participants in several sleep trajectory categories (eg, HRs for short-long trajectory, 1.22 [95% CI, 10.9-1.37] and 1.75 [95% CI, 1.50-2.03], respectively; *P* < .001), with greater risk observed among White participants (Figure 2A). The sleep trajectory and mortality association also significantly differed by household income of less than $15 000 and $15 000 or greater in the healthy-long (HRs, 1.09 [95% CI, 0.98-1.21] and 1.62 [95% CI, 1.43-1.84], respectively; *P* < .001), healthy-short (HRs, 1.06 [95% CI, 0.98-1.16] and 1.23 [95% CI, 1.11-1.37], respectively; *P* = .005), long-healthy (HRs, 1.16 [95% CI, 1.05-1.28] and 1.35 [95% CI, 1.18- 1.54], respectively; *P* = .01), long-long (HRs, 1.17 [95% CI, 1.03-1.33] and 1.54 [95% CI, 1.29-1.83], respectively; *P* = .002), short-healthy (HRs, 1.06 [95% CI, 0.99-1.14] and 1.19 [95% CI, 1.09-1.30], respectively; *P* = .02), and short-long (HRs, 1.26 [95% CI, 1.13-1.41] and 1.68 [95% CI, 1.43-1.98], respectively; *P* < .001) trajectories, with greater mortality risk observed among participants with income $15 000 or greater (Figure 2B). In the imputed datasets, the observed associations with all-cause mortality persisted for all suboptimal sleep trajectories except the short-short trajectory for model 4 (HR, 1.04; 95% CI, 0.98-1.09) (eTable 3 in Supplement 1). Excluding deaths within 2 years of follow-up did not substantially impact results (eTables 4 and 5 in Supplement 1). Excluding participants with a history of MI (eTable 6 in Supplement 1) or any history of MI, Parkinson disease, cancer, or stroke did not impact the direction or magnitude of the observed risks (healthy-long HR, 1.33 [95% CI, 1.09-1.61]; short-long HR, 1.26 [95% CI, 0.98-1.63] in model 3).

## Discussion

In a large representative cohort of Black and White middle-aged adults with lower household incomes and with repeated measures of sleep duration, an increased risk of all-cause and CVD mortality was observed among individuals with 5-year suboptimal sleep trajectories. Of note, compared with participants who maintained a healthy sleep duration of 7 to 9 hours, participants with short-long or long-short sleep trajectories were at a particularly greater mortality risk. These findings provide new evidence that irregular sleep patterns over a 5-year period may increase the risk of all-cause and CVD mortality. In race- and income-stratified analyses, associations were stronger among White compared with Black adults and among adults with a household income $15 000 or greater compared with adults with household incomes less than $15 000. No differences were observed by sex.

Our results support the current American Academy of Sleep Medicine and National Sleep Foundation recommendations for 7 to 9 hours of sleep per night for optimal health.^1,2^ Prior evidence connecting short and long sleep durations with adverse health outcomes, particularly cardiovascular risk,^10,12,13,25,26^ comes primarily from studies with sleep duration assessed at a single time point. It is not well understood how sleep duration changes over time throughout adulthood,^27^ and even less is known about how changes in sleep duration may be associated with mortality risk. In our cohort, we were surprised to find nearly two-thirds of participants had irregular or suboptimal sleep trajectories over the 5-year period. In the Whitehall II Study,^28^ 5-year changes in self-reported sleep duration were assessed among 5400 British working adults. Consistent with our sample, approximately one-third of the Whitehall II cohort maintained a sleep duration of 7 or 8 hours over the 5-year period.^28^ Further, indicators of SES, such as greater educational attainment and occupational position, were associated with optimal sleep durations.^28^ While the demographic composition of the cohorts differ, the proportion of adults who maintained optimal sleep duration over time and the observed differences in sleep trajectories by SES are consistent between cohorts. By leveraging repeated measures of sleep duration over a 5-year period, our study adds new evidence supporting the importance of investigating changes in sleep duration over time.

In our community-based cohort, suboptimal 5-year sleep trajectories were consistently associated with greater risk of all-cause and CVD mortality after adjustment for demographics, health behaviors, depressive symptoms, and comorbidities. Our findings are consistent in direction and magnitude with those reported in the Kailuan cohort among 52 599 Chinese adults,^29^ with sleep durations measured 3 times over a 4-year period. Suboptimal 4-year sleep trajectories were associated with increased risk of CVD events and all-cause mortality (HRs for mortality: 1.34 [95% CI, 1.15-1.57] for the normal-short trajectory, 1.50 [95% CI, 1.07-2.10] for the short-short trajectory).^29^ Our findings are also consistent with results from the Sleep Heart Health Study,^30^ in which 10-year sleep duration trajectories were assessed in a cohort of predominantly White older adults. In adjusted models, the short-long (HR, 1.74; 95% CI, 1.10-2.76), healthy-long (HR, 1.63; 95% CI, 1.26-2.13), and long-short (HR, 1.99; 95% CI, 1.03-4.05) trajectories were associated with a nearly 2-fold greater risk of all-cause mortality.^30^ These associations persisted after adjustment for important CVD risk factors. Similarly, when we adjusted for a range of comorbid conditions including traditional CVD risk factors, associations with all-cause and CVD mortality persisted across almost all suboptimal sleep trajectories, with greater risk observed particularly among the short-long and the long-short trajectories. Our findings are an important new contribution to the literature indicating that maintaining optimal sleep duration over a 5-year period may play an important role in reducing mortality risk.

We observed significant variations in the association between sleep trajectory and mortality by race and household income, but not sex. The observed associations appeared stronger among White compared with Black participants and among individuals with household incomes of $15 000 or greater compared with those with household incomes below $15 000. These findings were surprising considering SCCS participants with an optimal sleep trajectory were more likely to identify as White and have higher household incomes and given that the previous studies have shown that the association between sleep and CVD varies by race and income.^16^ Although the Black participants with low SES in the SCCS were more likely to have suboptimal sleep trajectories, our results suggest the potential health effects of suboptimal sleep may be weaker in these populations. This may be due in part to the presence of unmeasured cultural, behavioral, or societal factors (eg, racism or environmental factors) that may confound or attenuate the potential effect of sleep duration trajectory.^16^ Further, the fact that our results do not align with hypotheses and existing literature on sleep and health disparities highlights the challenge of using race in research, even as a social grouping category or proxy for other factors. Regardless, it is important to note that an association between suboptimal sleep trajectory and mortality was still observed among the Black participants and low-income subgroups, suggesting that sleep duration trajectory remained an important risk factor across all subgroups. While hypothesis generating, these results suggest there may be other factors, beyond the social constructs of race and income, that are not being captured and may negatively affect sleep and contribute to mortality.^31^ These results also highlight the importance of examining sleep in samples that offer more diverse representation not only in terms of race but also in neighborhood factors and SES.

We observed no consistent associations between suboptimal sleep trajectory and risk of cancer or neurodegenerative disease mortality. Previous findings on sleep duration and cancer mortality have been mixed, with some inconsistent evidence suggesting that long sleepers may be at greater mortality risk.^32^ Our null findings for neurodegenerative disease mortality may be due to the small number of deaths. The potential for underreporting of Alzheimer disease and Parkinson disease on death certificates has been documented,^33,34^ and this could contribute to misclassification bias limiting our ability to investigate potential associations with neurodegenerative disease mortality.

### Strengths and Limitations

A major strength of our study includes leveraging data from a large prospective cohort of Black and White individuals representing lower-income groups who have been historically underrepresented in other US cohorts. Participants reported sleep durations at 2 time points, and mortality data for all participants were obtained from NDI linkage.

Our study has several limitations to note. Sleep duration was the only component of sleep health measured in the SCCS. Thus, we are unable account for other potentially relevant dimensions of sleep health or sleep disorders, such as obstructive sleep apnea or insomnia, which may be important confounders. Further, we do not have information on sleep medication use, specifically sedatives or hypnotics. The measure used to assess sleep duration changed between the 2 time points. We explored the difference between the weighted and unweighted duration and found the difference insignificant. It is common for adults to overestimate their sleep duration,^35^ and we may have misclassified participants at either time point, which likely would bias our associations toward the null. There are limitations to NDI cause of death ascertainment, particularly for neurodegenerative disease, including inconsistencies in ordering cause of death across institutions and clinicians; however, the reported associations of sleep trajectory with all-cause mortality are not impacted by this potential bias.

## Conclusions

In this cohort study of Black and White adults with predominantly lower household incomes from the Southeastern US, suboptimal 5-year sleep duration trajectories were associated with greater risk of all-cause and CVD-specific mortality. Our study provides evidence that transitioning from short to long sleep duration (or the opposite) may increase the risk of death. Associations were observed among all subgroups; however, in stratified analyses, the HRs were greater among White participants and among those with a greater household income. Our findings support investigating changes in sleep duration as adults age and highlight the importance of maintaining healthy sleep durations throughout adulthood.

## Notes

Supplement 1. **eFigure 1.** SCCS Sleep Trajectory and Mortality Participant Flowchart **eFigure 2.** SCCS Sleep Trajectory and Mortality Analysis: Missing Data Distribution **eFigure 3.** Adjusted Survival Curves for All-Cause Mortality by Sleep Trajectory **eFigure 4.** Adjusted Hazard Ratios with 95% CIs for Each Cause-Specific Mortality by Sleep Trajectory Categories (Competing Risk Analysis) **eTable 1.** SCCS Participant Characteristics by Vital Status (N = 46 928) **eTable 2.** SCCS Sleep Trajectory and Mortality Analysis: Model Fit Metrics **eTable 3.** Associations Between Sleep Duration Trajectory and All-Cause Mortality in the Southern Community Cohort Study (MICE Imputed Dataset) **eTable 4.** Sleep Duration Trajectory and All-Cause Mortality in the SCCS Excluding Deaths Within the First 2 Years of Follow-Up **eTable 5.** Sleep Duration Trajectory and Cause-Specific Mortality in the SCCS Excluding Deaths Within the First 2 Years of Follow-Up **eTable 6.** Sleep Trajectory and All-Cause Mortality Excluding Participants With History of Myocardial Infarction (N = 41 555)

Supplement 2.
Data Sharing Statement
