# Supplementary material for: Sleep Trajectories and All-Cause Mortality Among Low-Income Adults
Source: JAMA Netw Open. 2025 Feb 27;8(2):e2462117. doi: 10.1001/jamanetworkopen.2024.62117 (PMC11868971; doi:10.1001/jamanetworkopen.2024.62117)
Supplement: Supplement 1. — eFigure 1. SCCS Sleep Trajectory and Mortality Participant Flowchart eFigure 2. SCCS Sleep Trajectory and Mortality Analysis: Missing Data Distribution eFigure 3. Adjusted Survival Curves for All-Cause Mortality by Sleep Trajectory eFigure 4. Adjusted Hazard Ratios with 95% CIs for Each Cause-Specific Mortality by Sleep Trajectory Categories (Competing Risk Analysis) eTable 1. SCCS Participant Characteristics by Vital Status (N = 46 928) eTable 2. SCCS Sleep Trajectory and Mortality Analysis: Model Fit Metrics eTable 3. Associations Between Sleep Duration Trajectory and All-Cause Mortality in the Southern Community Cohort Study (MICE Imputed Dataset) eTable 4. Sleep Duration Trajectory and All-Cause Mortality in the SCCS Excluding Deaths Within the First 2 Years of Follow-Up eTable 5. Sleep Duration Trajectory and Cause-Specific Mortality in the SCCS Excluding Deaths Within the First 2 Years of Follow-Up eTable 6. Sleep Trajectory and All-Cause Mortality Excluding Participants With History of Myocardial Infarction (N = 41 555) [file jamanetwopen-e2462117-s001.pdf]

Supplementary Online Content

Full KM, Shi H, Lipworth L, Dauer LT, Mumma MT, Xiao Q. Sleep trajectories and all-cause mortality among low-income adults. *JAMA Netw Open*. 2025;8(2):e2462117. doi:10.1001/jamanetworkopen.2024.62117

eFigure 1. SCCS Sleep Trajectory and Mortality Participant Flow Chart

eFigure 2. SCCS Sleep Trajectory and Mortality Analysis: Missing Data Distribution

eFigure 3. Adjusted Survival Curves for All-Cause Mortality by Sleep Trajectory

eFigure 4. Adjusted Hazard Ratios with 95% CIs for Each Cause-Specific Mortality by Sleep Trajectory Categories (Competing Risk Analysis)

eTable 1. SCCS Participant Characteristics by Vital Status (N = 46 928)

eTable 2. SCCS Sleep Trajectory and Mortality Analysis: Model Fit Metrics

eTable 3. Associations Between Sleep Duration Trajectory and All-Cause Mortality in the Southern Community Cohort Study (MICE Imputed Dataset)

eTable 4. Sleep Duration Trajectory and All-Cause Mortality in the SCCS Excluding Deaths Within the First 2 Years of Follow-Up

eTable 5. Sleep Duration Trajectory and Cause-Specific Mortality in the SCCS Excluding Deaths Within the First 2 Years of Follow-Up

eTable 6. Sleep Trajectory and All-Cause Mortality Excluding Participants With History of Myocardial Infarction (N = 41 555)

This supplementary material has been provided by the authors to give readers additional information about their work.

© 2025 Full KM et al. *JAMA Network Open*.

eFigure 1. SCCS Sleep Trajectory and Mortality Participant Flow Chart

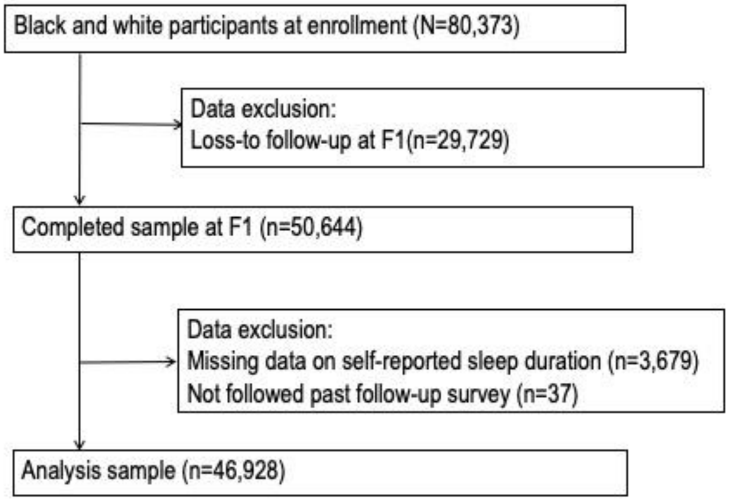

© 2025 Full KM et al. *JAMA Network Open*.

eFigure 2. SCCS Sleep Trajectory and Mortality Analysis: Missing Data Distribution

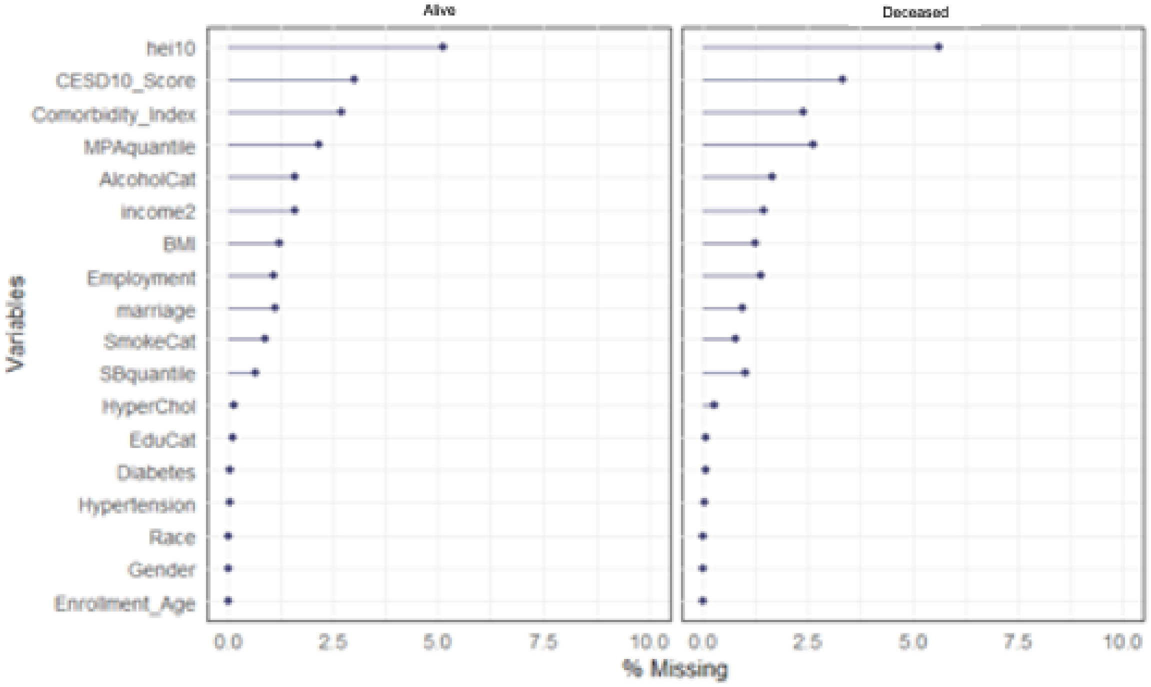

© 2025 Full KM et al. *JAMA Network Open*.

eFigure 3. Adjusted Survival Curves for All-Cause Mortality by Sleep Trajectory

A. Kaplan-Meier Plot without 95% Confidence Intervals

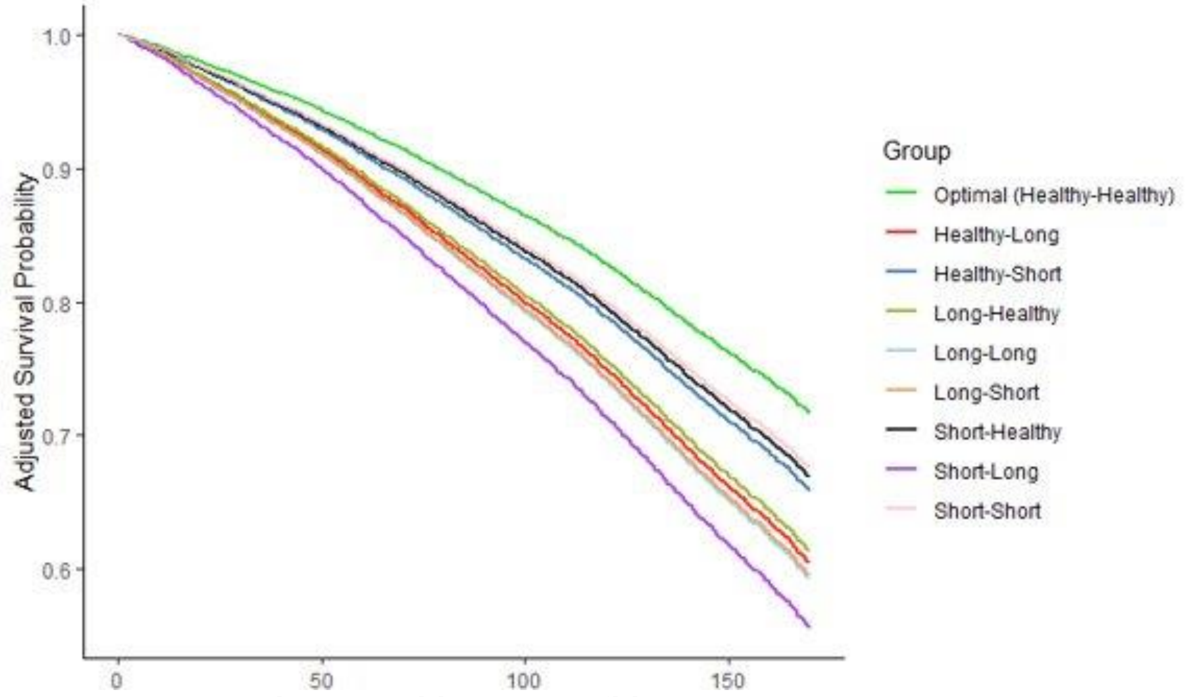

B. Kaplan-Meier Plot with 95% Confidence Intervals

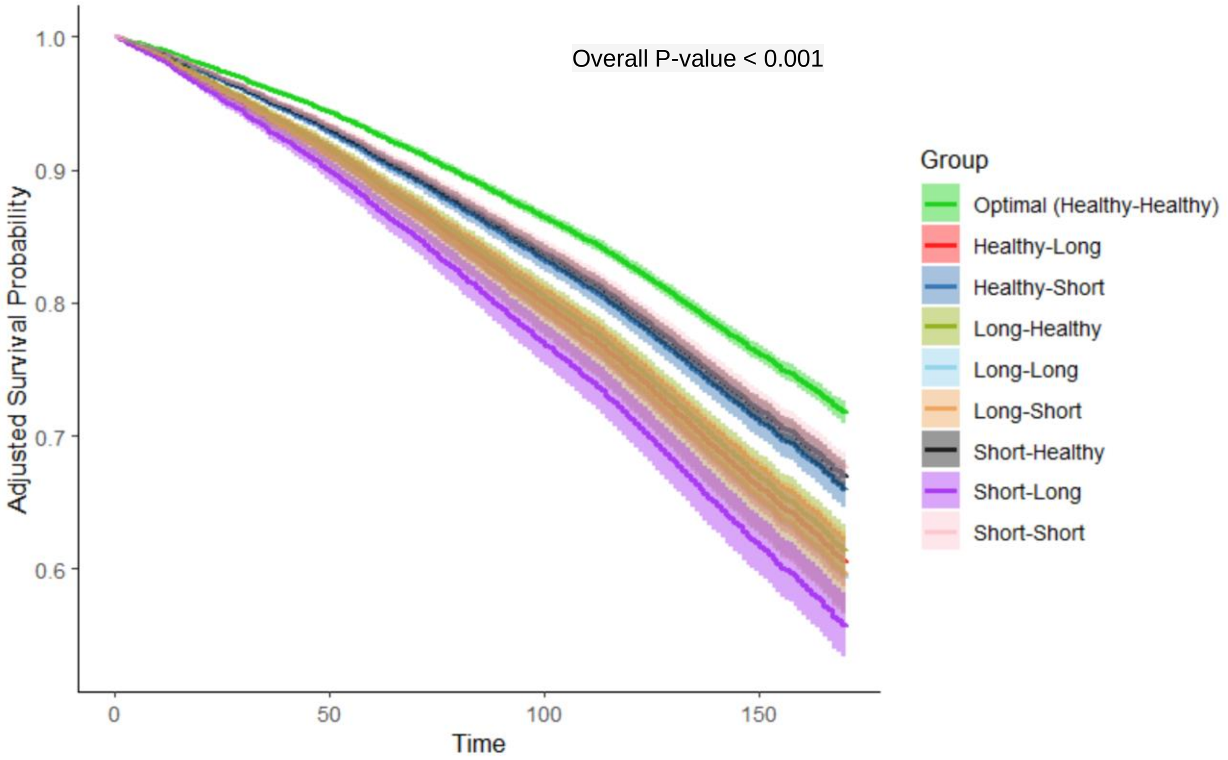

Adjusted for age, sex, and race.

© 2025 Full KM et al. *JAMA Network Open*.

eFigure 4. Adjusted Hazard Ratios with 95% CIs for Each Cause-Specific Mortality by Sleep Trajectory Categories (Competing Risk Analysis)

Cause-specific Mortality: Cancer Mortality; Neurodegenerative Disease Mortality; CVD Mortality

Sleep trajectories	Cancer cases	PDAD cases	CVD cases	HR (95% CI)
Optimal (Healthy-Healthy)	1013	220	1256	
Healthy-Long	192	35	253	1.21 (1.02, 1.43)<br>1.12 (0.77, 1.65)<br>1.18 (1.02, 1.37)
Healthy-Short	321	50	435	1.04 (0.90, 1.19)<br>1.00 (0.73, 1.38)<br>1.10 (0.97, 1.23)
Long-Healthy	190	28	271	1.17 (0.99, 1.38)<br>1.08 (0.69, 1.69)<br>1.24 (1.07, 1.43)
Long-Long	93	15	142	1.05 (0.83, 1.33)<br>1.24 (0.71, 2.16)<br>1.29 (1.07, 1.56)
Long-Short	60	7	118	0.90 (0.68, 1.20)<br>0.83 (0.36, 1.87)<br>1.40 (1.14, 1.71)
Short-Healthy	506	86	695	1.02 (0.91, 1.14)<br>1.06 (0.81, 1.39)<br>1.12 (1.01, 1.24)
Short-Long	111	24	187	0.98 (0.79, 1.21)<br>1.47 (0.93, 2.34)<br>1.32 (1.12, 1.57)
Short-Short	581	79	778	1.00 (0.90, 1.12)<br>0.95 (0.72, 1.26)<br>1.09 (0.99, 1.20)

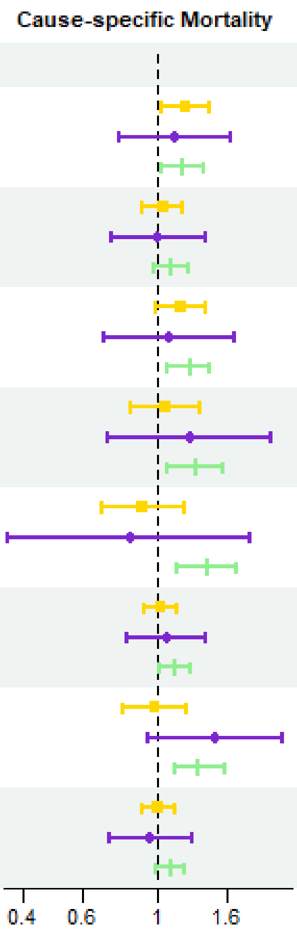

Adjusted for age, sex, race, education, marital status, household income, employment status, smoking status, alcohol intake, HEI-2010, total moderate activity, sedentary time.

© 2025 Full KM et al. *JAMA Network Open*.

eTable 1. SCCS Participant Characteristics by Vital Status (N = 46 928)

	Overall N = 46,928	Alive N = 33,349	Deceased N = 13,579	p-value*
Age, years	53.0 ± 8.8	51.6 ± 8.0	56.5 ± 9.6	<0.001
Women	30,712 (65.4)	22,911 (68.7)	7,801 (57.4)	<0.001
Black adults	29,725 (63.3)	21,320 (63.9)	8,405 (61.9)	<0.001
Less than high school	11,129 (23.7)	6,838 (20.5)	4,291 (31.6)	<0.001
Married	18,969 (40.9)	14,116 (42.8)	4,853 (36.1)	<0.001
Household income > $15k	23,911 (51.7)	18,505 (56.4)	5,406 (40.4)	<0.001
Unemployed	26,158 (56.4)	16,486 (50.0)	9,672 (72.2)	<0.001
Current smoker	15,739 (33.8)	10,150 (30.7)	5,589 (41.5)	<0.001
Alcohol consumption, ≥1 drink/day	8,252 (17.9)	5,736 (17.5)	2,516 (18.8)	<0.001
HEI-2010	58.8 (50.2, 67.7)	59.0 (50.3, 67.9)	58.4 (49.9, 67.1)	<0.001
physical activity, MET-hour/day	8.0 (4.0, 13.1)	8.0 (4.0, 13.7)	6.9 (2.9, 12.0)	<0.001
Sitting time, hrs/day	8.7 (6.0, 12.0)	9.0 (6.0, 12.0)	8.5 (6.0, 12.0)	<0.001
BMI, kg/m^2	29.6 (25.5, 35.0)	29.6 (25.6, 34.8)	29.6 (25.1, 35.4)	0.004
Hypertension	26,337 (56.1)	17,151 (51.4)	9,186 (67.7)	<0.001
Diabetes	10,072 (21.5)	5,571 (16.7)	4,501 (33.2)	<0.001
High cholesterol	17,598 (37.6)	11,829 (35.5)	5,769 (42.6)	<0.001
MI at enrollment	3,151 (6.7)	1,433 (4.3)	1,718 (12.7)	<0.001
MI at follow-up	1,926 (4.2)	922 (2.8)	1,004 (7.6)	<0.001
Sleep trajectory				<0.001
Optimal (healthy-healthy)	15,781 (33.6)	11,584 (34.7)	4,197 (30.9)	
Long-Long	1,283 (2.7)	826 (2.5)	457 (3.4)	
Long-Healthy	2,581 (5.5)	1,732 (5.2)	849 (6.3)	
Long-Short	990 (2.1)	655 (2.0)	335 (2.5)	
Healthy-Long	2,401 (5.1)	1,539 (4.6)	862 (6.3)	
Healthy-Short	4,900 (10.4)	3,461 (10.4)	1,439 (10.6)	
Short-Long	1,628 (3.5)	1,020 (3.1)	608 (4.5)	
Short-Healthy	7,878 (16.8)	5,597 (16.8)	2,281 (16.8)	
Short-Short	9,486 (20.2)	6,935 (20.8)	2,551 (18.7)	

Data presented as mean ±SD, Median (IQR) or Frequency (%).
*Wilcoxon rank sum test; Pearson's Chi-squared test

© 2025 Full KM et al. *JAMA Network Open*.

eTable 2. SCCS Sleep Trajectory and Mortality Analysis: Model Fit Metrics

Model	AIC	BIC	C-Index	χ^2 (p value)
Model 1	282128.3	282211.0	0.6484145	
Model 2	268706.9	268819.1	0.6855002	M1- M2: 1701.1 (<0.005)
Model 3	241995.1	242172.4	0.6979306	M2 – M3: 707.53 (<0.005)
Model 4	230338.6	230536.9	0.7103986	M3 – M4: 749.15 (<0.005)

© 2025 Full KM et al. *JAMA Network Open*.

eTable 3. Associations Between Sleep Duration Trajectory and All-Cause Mortality in the Southern Community Cohort Study (MICE Imputed Dataset)

Sleep trajectory	Model 1[a] HR (95% CI)	Model 2[b] HR (95% CI)	Model 3[c] HR (95% CI)	Model 4[d] HR (95% CI)
Optimal (Healthy-Healthy)	—	—	—	—
Long-Long	1.61 (1.44, 1.80)	1.35 (1.21, 1.51)	1.28 (1.14, 1.43)	1.23 (1.11, 1.35)
Long-Healthy	1.50 (1.40, 1.62)	1.31 (1.21, 1.41)	1.24 (1.15, 1.34)	1.21 (1.12, 1.30)
Long-Short	1.61 (1.44, 1.80)	1.35 (1.21, 1.51)	1.27 (1.14, 1.43)	1.21 (1.08, 1.36)
Healthy-Long	1.56 (1.45, 1.67)	1.33 (1.23, 1.43)	1.30 (1.20, 1.40)	1.26 (1.16, 1.36)
Healthy-Short	1.27 (1.20, 1.35)	1.16 (1.09, 1.24)	1.15 (1.08, 1.22)	1.13 (1.06, 1.20)
Short-Long	1.83 (1.68, 2.00)	1.48 (1.36, 1.62)	1.44 (1.32, 1.57)	1.35 (1.23, 1.48)
Short-Healthy	1.22 (1.16, 1.29)	1.16 (1.10, 1.22)	1.14 (1.08, 1.20)	1.09 (1.04, 1.16)
Short-Short	1.23 (1.13, 1.25)	1.12 (1.06, 1.18)	1.10 (1.04, 1.15)	1.04 (0.98, 1.09)

HR = Hazard Ratio, CI = Confidence Interval; [a]Model 1: adjusted for age, sex, race; [b]Model 2: model 1 + adjustment for education, marital status, household income, employment status; [c]Model 3: model 2 + adjustment for smoking status, alcohol intake, HEI-2010, total moderate activity, sedentary time; [d]Model 4: model 3 + adjustment for BMI, CCI, CES-D Score

© 2025 Full KM et al. *JAMA Network Open*.

eTable 4. Sleep Duration Trajectory and All-Cause Mortality in the SCCS Excluding Deaths Within the First 2 Years of Follow-Up

Sleep trajectory	N	Events N	Model 1[a] HR (95% CI)	Model 2[b] HR (95% CI)	Model 3[c] HR (95% CI)	Model 4[d] HR (95% CI)
Optimal (Healthy-Healthy)	15,387	3,803	—	—	—	—
Long-Long	1,226	400	1.58 (1.43, 1.75)	1.29 (1.17, 1.44)	1.28 (1.15, 1.44)	1.25 (1.12, 1.40)
Long-Healthy	2,512	780	1.54 (1.43, 1.67)	1.33 (1.23, 1.44)	1.29 (1.19, 1.41)	1.23 (1.13, 1.34)
Long-Short	951	296	1.58 (1.40, 1.78)	1.31 (1.16, 1.48)	1.23 (1.08, 1.40)	1.16 (1.02, 1.33)
Healthy-Long	2,281	742	1.50 (1.38, 1.62)	1.28 (1.18, 1.38)	1.22 (1.12, 1.33)	1.17 (1.07, 1.27)
Healthy-Short	4,737	1,276	1.25 (1.17, 1.33)	1.14 (1.06, 1.21)	1.12 (1.04, 1.20)	1.09 (1.02, 1.17)
Short-Long	1,548	528	1.79 (1.63, 1.96)	1.44 (1.31, 1.58)	1.38 (1.25, 1.52)	1.27 (1.15, 1.40)
Short-Healthy	7,640	2,043	1.21 (1.15, 1.28)	1.14 (1.08, 1.20)	1.12 (1.06, 1.19)	1.07 (1.01, 1.14)
Short-Short	9,215	2,280	1.18 (1.12, 1.24)	1.11 (1.05, 1.17)	1.08 (1.02, 1.14)	1.01 (0.95, 1.07)

HR = Hazard Ratio, CI = Confidence Interval; [a]Model 1: adjusted for age, sex, race; [b]Model 2: model 1 + adjustment for education, marital status, household income, employment status; [c]Model 3: model 2 + adjustment for smoking status, alcohol intake, HEI-2010, total moderate activity, sedentary time; [d]Model 4: model 3 + adjustment for BMI, CCI, CES-D Score

© 2025 Full KM et al. *JAMA Network Open*.

eTable 5. Sleep Duration Trajectory and Cause-Specific Mortality in the SCCS Excluding Deaths Within the First 2 Years of Follow-Up

	Event	Model 1 HR (95% CI)		Model 2 HR (95% CI)		Model 3 HR (95% CI)		Model 4 HR (95% CI)	
CVD Mortality									
	3,693								
Healthy-Healthy	1,129	—	—	—	—	—	—	—	—
Long-Long	122	1.51	1.25, 1.83	1.27	1.05, 1.54	1.26	1.03, 1.54	1.18	0.96, 1.46
Long-Healthy	246	1.49	1.29, 1.72	1.28	1.11, 1.48	1.26	1.08, 1.47	1.17	1.00, 1.37
Long-Short	100	1.61	1.31, 1.97	1.35	1.10, 1.67	1.31	1.05, 1.63	1.22	0.97, 1.53
Healthy-Long	215	1.31	1.13, 1.52	1.15	0.99, 1.34	1.13	0.96, 1.32	1.09	0.93, 1.28
Healthy-Short	393	1.22	1.09, 1.37	1.12	0.99, 1.26	1.11	0.98, 1.26	1.07	0.94, 1.21
Short-Long	162	1.65	1.39, 1.95	1.37	1.15, 1.62	1.32	1.11, 1.58	1.22	1.02, 1.47
Short-Healthy	626	1.21	1.10, 1.34	1.14	1.03, 1.26	1.12	1.01, 1.25	1.08	0.97, 1.20
Short-Short	700	1.18	1.08, 1.30	1.11	1.01, 1.23	1.10	0.99, 1.21	1.02	0.92, 1.13
Cancer Mortality									
	2,655								
Healthy-Healthy	889	—	—	—	—	—	—	—	—
Healthy-Long	148	1.16	0.97, 1.38	1.08	0.90, 1.29	1.07	0.89, 1.30	1.01	0.83, 1.24
Healthy-Short	279	1.11	0.97, 1.27	1.05	0.91, 1.20	1.02	0.88, 1.19	1.02	0.88, 1.18
Long-Healthy	172	1.31	1.11, 1.54	1.23	1.04, 1.46	1.20	1.00, 1.43	1.19	1.00, 1.43
Long-Long	75	1.15	0.91, 1.46	1.05	0.82, 1.33	1.00	0.77, 1.29	0.99	0.76, 1.28
Long-Short	53	1.08	0.82, 1.44	1.00	0.75, 1.33	0.95	0.70, 1.28	0.95	0.70, 1.30
Short-Healthy	437	1.06	0.95, 1.19	1.03	0.92, 1.16	1.02	0.90, 1.15	1.01	0.89, 1.14
Short-Long	93	1.16	0.93, 1.44	1.01	0.81, 1.26	0.97	0.76, 1.22	0.94	0.74, 1.20
Short-Short	509	1.07	0.96, 1.20	1.06	0.94, 1.18	1.02	0.90, 1.15	1.01	0.89, 1.14
Neurodegenerative Disease Mortality									
	530								
Healthy-Healthy	214	—	—	—	—	—	—	—	—
Healthy-Long	33	1.10	0.76, 1.60	1.07	0.73, 1.58	1.12	0.75, 1.67	1.04	0.68, 1.58
Healthy-Short	49	0.97	0.71, 1.32	0.98	0.71, 1.34	1.03	0.74, 1.42	1.02	0.73, 1.42
Long-Healthy	27	1.19	0.79, 1.79	1.07	0.70, 1.65	1.04	0.66, 1.65	1.06	0.67, 1.69
Long-Long	--	1.28	0.75, 2.17	1.30	0.76, 2.22	1.33	0.76, 2.33	1.31	0.73, 2.34
Long-Short	--	--	--	--	--	--	--	--	--
Short-Healthy	83	1.02	0.79, 1.32	1.00	0.77, 1.31	1.06	0.81, 1.39	1.02	0.77, 1.37
Short-Long	24	1.73	1.12, 2.67	1.59	1.01, 2.50	1.60	1.01, 2.55	1.59	0.99, 2.54
Short-Short	78	0.93	0.72, 1.21	0.96	0.74, 1.26	0.97	0.73, 1.28	0.97	0.72, 1.30

HR = Hazard Ratio, CI = Confidence Interval; Model 1: adjusted for age, sex, race; Model 2: model 1 + adjustment for education, marital status, household income, employment status; Model 3: model 2 + adjustment for smoking status, alcohol intake, HEI-2010, total moderate activity, sedentary time; Model 4: model 3 + adjustment for BMI, CCI, CES-D Score

© 2025 Full KM et al. *JAMA Network Open*.

eTable 6. Sleep Trajectory and All-Cause Mortality Excluding Participants With History of Myocardial Infarction (N = 41 555)

Sleep trajectory	N	Events N	Model 1[a] HR (95% CI)	Model 2[b] HR (95% CI)	Model 3[c] HR (95% CI)	Model 4[d] HR (95% CI)
Optimal (Healthy-Healthy)	14,231	3,424	—	—	—	—
Long-Long	1,124	375	1.66 (1.49, 1.85)	1.34 (1.20, 1.50)	1.35 (1.20, 1.51)	1.30 (1.16, 1.47)
Long-Healthy	2,243	674	1.50 (1.38, 1.63)	1.30 (1.20, 1.42)	1.26 (1.15, 1.38)	1.22 (1.11, 1.33)
Long-Short	865	264	1.57 (1.38, 1.78)	1.30 (1.14, 1.48)	1.23 (1.07, 1.40)	1.18 (1.02, 1.35)
Healthy-Long	2,077	683	1.57 (1.45, 1.71)	1.33 (1.22, 1.44)	1.28 (1.17, 1.40)	1.23 (1.12, 1.35)
Healthy-Short	4,382	1,182	1.27 (1.19, 1.36)	1.17 (1.09, 1.25)	1.14 (1.06, 1.22)	1.11 (1.04, 1.20)
Short-Long	1,379	474	1.84 (1.67, 2.02)	1.46 (1.33, 1.62)	1.40 (1.26, 1.55)	1.30 (1.17, 1.45)
Short-Healthy	6,946	1,814	1.20 (1.13, 1.27)	1.13 (1.07, 1.20)	1.12 (1.06, 1.19)	1.08 (1.02, 1.15)
Short-Short	8,308	2,010	1.17 (1.10, 1.23)	1.10 (1.04, 1.17)	1.07 (1.01, 1.13)	1.02 (0.95, 1.08)

HR = Hazard Ratio, CI = Confidence Interval; [a]Model 1: adjusted for age, sex, race; [b]Model 2: model 1 + adjustment for education, marital status, household income, employment status; [c]Model 3: model 2 + adjustment for smoking status, alcohol intake, HEI-2010, total moderate activity, sedentary time; [d]Model 4: model 3 + adjustment for BMI, CCI, CES-D Score

© 2025 Full KM et al. *JAMA Network Open*.
