# Supplementary material for: Sleep Trajectories and All-Cause Mortality Among Low-Income Adults
Source: JAMA Netw Open. 2025 Feb 27;8(2):e2462117. doi: 10.1001/jamanetworkopen.2024.62117 (PMC11868971; doi:10.1001/jamanetworkopen.2024.62117)
Supplement: Supplement 2. — Data Sharing Statement [file jamanetwopen-e2462117-s002.pdf]

Data Sharing Statement

Full. Sleep Trajectories and All-Cause Mortality Among Low-Income Adults. *JAMA Netw Open*. Published February 27, 2025. doi:10.1001/jamanetworkopen.2024.62117

Data
Data available: Yes
Data types: Deidentified participant data, Data dictionary
How to access data: https://www.southerncommunitystudy.org/research-opportunities.html
When available: With publication

Supporting Documents
Document types: None

Additional Information
Who can access the data: researchers whose proposed use of the data has been approved by the SCCS.
Types of analyses: for any purpose that is approved by SCCS
Mechanisms of data availability: after approval of a proposal and a subsequent signed data access agreement
